# "Placebo effect is probably what we refer to as patient healing power": A qualitative pilot study examining how Norwegian complementary therapists reflect on their practice

**DOI:** 10.1186/s12906-017-1770-8

**Published:** 2017-05-12

**Authors:** Trine Stub, Nina Foss, Ingrid Liodden

**Affiliations:** 1 The National Research Center in Complementary and Alternative Medicine (NAFKAM) Department of Community Medicine, Faculty of Health Sciences, UiT, The Arctic University of Norway, Tromsø, Norway; 2 Department of Nursing, Faculty of Health Sciences, Oslo and Akershus University College of Applied Science (HiOA), Oslo, Norway

**Keywords:** Consultation, Placebo, Communication, Complementary therapies, Contextual healing

## Abstract

**Background:**

Complementary therapists spend considerable time with their patients, especially in the first consultation. The communication between patients and their therapists is important for raising consciousness and activation of the patient’s self-healing power. Thus, the aims in this study were to delineate what complementary therapists regard as essential in patient consultations, their view of the healing process, and how the therapists understand the placebo effect and its position in the healing process.

**Methods:**

Semi-structured individual interviews (*n* = 4), focus group interview (*n* = 1) and participant observation were conducted among four different complementary therapists in a Norwegian community. The text data was transcribed verbatim and the analysis of the material was conducted according to conventional and direct content analysis. Some codes were predefined and others were defined during the analysis.

**Results:**

The pilot study showed that the implemented methods seems feasible and fit well with the aims of this study. Complementary therapists (chiropractor, naprapath (musculoskeletal therapist), acupuncturist and acupuncturist/homeopath) representing four different complementary modalities participated. A combination of the conversation and examination during the first consultation formed the basis for the therapist’s choice of treatment. A successful consultation was characterized by a fruitful relationship between the therapist and the patient. Moreover, the therapist needs to be humble and show the patient respect. Patients’ positive beliefs and expectations about the treatment play a significant role in the healing process. The more hope the therapist can bring about, the more easily the patient can start believing that it is possible to get well.

**Conclusion:**

This was a pilot study. Therefore the findings should be appreciated as limited and preliminary. Therapists’ and patients’ mutual understanding and treatment goals were essential for a successful consultation. The therapists emphasized their professional skills and therapeutic competence as important when building fruitful relationships with their patients. Exerting authority and making the patient feel confident were essential factors for a successful healing process. The complementary therapists understood the placebo effect as the patient’s self-healing power, resulting from establishing trust and belief in the treatment process.

**Electronic supplementary material:**

The online version of this article (doi:10.1186/s12906-017-1770-8) contains supplementary material, which is available to authorized users.

## Background

The establishment and maintenance of sound relations, and the communication between the therapists and patients are of great importance in all complementary therapies. A common basic thought in most modalities included in complementary therapies is that the therapists’ main aim is to strengthen the patient’s self-healing power [1, 2]. This is achieved through various therapeutic interventions characteristic for each modality, for example chiropractic, homeopathy, acupuncture, acupressure, and the use of herbs and dietary supplements. The various complementary therapies stress the specific effect to different degrees as well as the non-specific effects (placebo) of the interventions on the patient’s self-healing power [3]. However, all modalities emphasize the importance of consultations [4]. They are essential to enable determination of diagnoses and interventions. The communication between patients and therapists is also essential in raising consciousness and activate the patient’s self-healing power. The patient’s self-healing power comes from the ancient tradition named *resonance* understood as an elicitation of inner potential to recreate balance [5].

Complementary therapists spend considerable time with their patients, especially in the first consultation [6]. Studies demonstrate that the patients’ first consultations with complementary therapists last longer than equivalent consultations with conventional providers [7]. They emphasize the importance of collecting information about the patients’ previous and current life situation in terms of issues, such as diets, emotional and physical stress, and the management of possible problems and traumas [8]. Furthermore, complementary therapists stress the importance of showing empathy, trust building, and care (giving) [8, 9].

A study of the patients’ experiences in complementary consultations demonstrates that patients feel connected to the therapists, and that they are seen and heard as whole persons [8, 10]. In addition, patients value the holistic approach, and they find these consultations empowering, enabling them to learn more about their own health [11–13]. Moreover, studies investigating patients’ satisfaction with complementary therapies generally reveal great satisfaction, and this is strongly connected to how the patients experience the consultations [2, 8, 14]. Studies in conventional medicine show similar connections between caring, empathic therapy and improved health effect for the patients [15–17].

### Placebo effect

Placebos are physical interventions that are administrated in the guise of an active treatment, but do not have the physical properties ascribed to them [18]. A placebo effect should be elicited in any clinical practice to optimize the effect of the treatment. Furthermore, placebo effects are recognized to be present in all clinical encounters despite attempts to control them [19].

Placebo effects may be defined as “a beneficial effect in a patient following a particular treatment that arises from the patient expectancy concerning the treatment rather than from the treatment itself” [20]. It is typically held that expectancy does not alone account for all placebo effects [21], but it is probably the single most important factor involved [22]. Thus, the patients’ positive attitudes and confidence in benefit from a treatment are shown to produce placebo effects. In clinical studies seeking to understand therapeutic processes as complex healing, placebo effects have been connected to the patients’ expectations of the therapy, the relation between the therapists and patients, and to the socio-cultural meaning of the therapy. Thus, the patients’ positive attitudes and confidence benefit from a fruitful relationship between the therapists and patients [23]. An alternative way of conceiving the placebo effect is contextual healing, understood in this paper as the aspect of healing that is produced by the context of the consultation, as separated from the specific efficacy of the treatment interventions [24].

### Theoretical approach

When observing consultations, some perspectives on functions and phases in the consultations may contribute to constructive focus. Kringlen and Finset [17] provide a model for clinical consultation that is divided into three functions: 1) map the patient’s problem, 2) maintain the relation between the therapist and patient, and 3) inform and initiate treatment. They connect 1) and 3) to task-oriented aspects, and 2) to emotional aspects. Based on research, they argue that patient-oriented medicine should maintain task-oriented as well as emotional aspects. This may be related to the difference between “curing” and “healing”. “Curing refers to an act of treating successfully a specific condition […] Healing, by contrast, refers to the whole person, or the whole body seen as an integrated system with both physical and spiritual components” [25]. Strathern and Steward [25] claim that biomedicine is primarily concerned with curing, whereas complementary modalities belong to the treatment philosophy of healing, in which some incorporate curing, and others do not. According to Helman [23] curing and healing are both included in practical clinical work in all medical systems. Clinical consultations in conventional as well as complementary modalities may also be regarded as healing rituals. Rituals and symbolic aspects of consultations support confidence in the therapist and valid treatment, which contribute to healing [23]. In a systematic review, Di Blasi et al. [14] investigated contextual effects of health in consultations between physicians and patients. They argued for more focus on research in the human sides of the treatment to achieve better quality in the health services. Furthermore, they emphasized the importance of studying the interaction between therapists and patients in conventional treatment as well as in complementary therapies. This needs to be explored through qualitative as well as quantitative approaches. In the pilot study presented here, we will delineate what complementary therapists regard as essential in patient consultations and how the patient’s self-healing power can be stimulated. We will also assess the feasibility of methods and procedures applied for later use in a larger study. Hence the aims of this study are: To map what complementary therapists consider essential in their consultation. We will look for their views of the healing processes, and what they find important in interacting with their patients during consultations.

To gain preliminary knowledge about how complementary therapists understand the placebo effect and its position in the healing process.

## Methods

### Design

In this study we applied a design based on social qualitative fieldwork. Fieldwork is intense, long term anthropological research conducted among a community of people. The design aims to gain a close and intimate familiarity with a given group of individuals and their practices through intensive involvement with people in their cultural environment [26]. Qualitative research focuses on the content, nature and meaning [27]. Qualitative design may also contribute to a better understanding of important issues in health and well-being [28]. In complementary therapies qualitative methodology is fundamental to understand key treatment components, the philosophical basis and contextual frameworks of these modalities [4, 29]. We have studied natural processes in limited social situations using participant observation, semi-structured interviews and focus group interviews. We intended to understand how complementary consultations are practiced and how the therapists experience their own practices of patient consultations.

### Study area

The study location was a community in Telemark county with 15,000 inhabitants. The study took place in an alternative and complementary outpatient clinic, where four therapists worked in neighboring offices on a daily basis. In the course of one week during autumn 2013, the complementary therapists representing four different complementary modalities (chiropractor, naprapath (musculoskeletal therapist), acupuncturist and acupuncturist/homeopath) were observed during their consultations with patients. Following the observations, all four therapists were interviewed individually by using semi-structured interviews. On the last day of the observation period all four therapists participated in a focus group interview.

### Participants

According to a Norwegian study from 2014 [30] the three most popular complementary modalities used by Norwegian patients were massage therapy (17.2%), acupuncture (6.9%) and naprapathy (musculoskeletal therapy) (4.2%). This information was the rationale for choosing these particular study participants.

We included complementary therapists, three (*n* = 3) females and one male (*n* = 1) (Table 1). They were trained in different complementary therapies and worked in the same clinic. They had all more than five years of experience in their professional fields, and all but one offered additional therapies, such as dietary supplement and exercise advice. Three (*n* = 3) worked full time and one (*n* = 1) worked part time. Two (*n* = 2) had been working in clinical practice between 5 to 9 years and two (*n* = 2) had been working for more than 20 years. The first consultation varied from 30 to 60 min, and the therapists often offered their patients additional therapies.

**Table 1:** Characteristics of complementary therapists

Full time chiropractor	1
Full time acupuncturist	2
Full time naprapath	1
Part time homeopath and acupuncturist	1
Years of practice	
0–4 years	
5–9 years	2
10–14 years	
15–19 years	
20–24 years	1
25–29 years	
More than 30 years	1
Length of initial consultation	
Approximately 30 min	1
Approximately 60 min	3
Additional therapies	
Vitamins/minerals/herbs	3
Gender	
Female	3
Male	1

### Participant observation

Participant observation is a method to collect data that involves the researcher to participate in the social processes of the studies. The focus is on present natural processes in limited parts of social life [31–33]. In this study the focus was on what the study participants consider important in their consultation and their interaction with the patients. During the consultations the observer sat on a chair in the consultation room, where she took notes and listened to the conversation between the patient and the therapist. When the patient lay on the bench, she stood beside the bench to observe what happened. If something was unclear, she asked the therapist to clarify. When the therapists were asked how the observer’s presence affected the consultations, they answered that her presence made no difference because they had scheduled for less demanding patients that day, to have time for the observer. Data gathered from these observations formed the basis for preparation and conduct of the individual and group interviews and was included in the content analysis.

### Individual semi-structured interviews

The interviews were semi-structured and based on an interview guide developed by two of the investigators (TS and NF), based on their own understanding and knowledge from existing literature on complementary modalities. Semi-structured interviews are used when trying to understand daily life based on the participants’ own perspective and lifeworld [34]. Such interviews make it possible to acquire new knowledge about specific issues through allowing questions to be created during the interview, in addition to pre-defined themes or questions from the interview guide [34]. The interview guide was also based on Kleinman’s explanatory model concept [35]. The interview guide included the following main questions: *How did you experience the therapy today? / Differences and similarities between initial and later consultations?* / *What are the most common problems for your patients? / Can you describe important principles of good consultations?* / *Which competence is the most important for a therapist?* / *What is essential to stimulation of the patients*’ *self-healing power? / What is the relation between symptomatic and comprehensive treatment?* / *What are your strengths as a therapist?* (The interview guide is attached as Additional file 1.) One of the researchers (NF) guided the individual interviews and led the focus group interview.

### Focus group interviews

Focus group interviews generate different types of knowledge compared to individual interviews, as the group dynamics may reveal issues and reflections of individual practice and experience that will not be disclosed in individual interviews [36]. Focus group interviews are beneficial when the aim is to understand what complementary therapists experience (is) as essential in their own consultation practice [37]. Concrete questions for the focus group interviews were developed based on the findings in the observations, reflections and semi-structured interviews. The interview lasted for an hour and was conducted at the clinic during an extended lunch break.

### Data analysis

We used qualitative content analysis to analyze the text data focusing on the characteristics of language as a means of communication addressing the content or contextual meaning. We wanted to provide knowledge of the phenomenon under investigation through a systematic classification process of coding and identifying themes [38]. The analysis of our material was conducted according to conventional and direct content analysis. Conventional content analysis is generally used to describe a phenomenon and is appropriate when existing theory or research literature on a phenomenon is limited. A directed approach to content analysis is useful when existing theory or prior research exist, but the information is incomplete or would benefit from further description.

Some codes were predefined and others were defined during the analysis [38, 39]. Codes were grouped according to the eight questions in the interview guide. Groups with overlapping information were merged into five main themes. The themes were renamed according to key information in the text data [40]. The consultations were recorded on tape, anonymized and transcribed. To ensure trustworthiness, we coded directly from the text data and employed words and phrases used by the participants during the interviews when analyzing. Two of the authors (TS and IL) read the material several times and discussed the various steps of the analysis process, and the third author (NF), who performed the interviews, read and accepted the result. The quotations were printed in Norwegian before being translated into English.

## Results

There were no difficulties in recruiting participants, and all but one patient allowed the observer access to the consultation room. Four complementary therapists representing different complementary modalities (chiropractor, naprapath (musculoskeletal therapist), acupuncturist and acupuncturist/homeopath) participated in the study. When performing the data analysis, we focused on what complementary therapists regard as essential in patient consultations, their views on the healing process, and how the patient’s self-healing power can be stimulated. Five themes arose from our content analysis: 1) Patients’ common health issues, 2) The initial and follow-up consultations, 3) Good consultations are characterized by building trust, 4) Healing hands and humour 5) Stimulation of the patient’s self-healing power.

### Patients’ common health issues

Common health issues of patients visiting the clinic are characterized by the therapists as indecisive and subjective symptoms.

A: Anxiety and stress, musculoskeletal pain and menopause problems, such as sleeping problems, restless legs and hot flashes.

J: A lot of general muscle pain and headache, numbness in fingers and cramps.

C: That’s everything that doesn’t show on clinical tests. They get much help from Western medicine and their physician. But then there are all the unspecific issues. All the things that have not yet become a physical disease, the imbalance that makes people feel ill, that doesn’t expose high levels of CRP,^1^ for example.

Patients mostly seek help for different unspecific symptoms. Specific diseases that can be diagnosed in clinical laboratory tests are typically treated by their physicians. Many patients have been or are simultaneously treated by the conventional health care system.

### The initial and follow-up consultations

All therapists agreed that the first consultation is important for establishing trust and good relationships with the patients. The initial consultation typically lasts for an hour. The main focus initially is a thorough mapping of the patients’ complaints and symptoms through interviews and examinations. The initial consultation is extensive, starting with an interview and physical examination. The focus is on symptoms - potential patterns of symptoms, observations, diagnoses, and treatment plan.

The combination of the conversation and examination during the initial consultation provides a picture of the patient’s complaints that form the basis for the therapist’s choice of treatment. The consultation is followed by a short treatment, ensuring that the first session does not become too excessive for the patients. Of importance is to build a relationship with the patient based on confidence in the therapist and beliefs about the effectiveness of the treatment.

S: [In the initial consultation] we look deeper into the complaints. The patients often tell how it started, where it hurts, what makes it hurt, or what makes it worse, and what makes it better. I ask many questions about the complaints. The actual treatment is more lenient and shorter the first time as we’ve experienced that the patients may react somewhat differently. Some may get a lot worse. Others don’t have worsening of the symptoms afterwards. However, to be on the safe side, I don’t do too much the first time, just to see how they react.

C: My focus is much on the personality of the patient. I try to find out what the patient thinks about the cause of the illness. I ask many questions and try to understand why the patient has become ill. You often need three to four key symptoms that fit a particular homeopathic remedy. So you have to dig deep. You have to ask time and again till you get a clear picture of the symptoms and the underlying causes, and try to map any relations, either in the family or elsewhere, that may affect the patient.

J: Trust is vital. You have to be able to understand the patient. You can’t treat all patients equally, that won’t work. You have to adapt and render trust.

The follow-up consultations last shorter, and the therapist’s emphasis is more on the treatment per se. The therapist may gradually look deeper into personal issues.

S: I may focus more on that [personal issues, such as stress, psychological problems and relations to family and friends] in the next consultation, if they’ve had severe reactions, or if I sense that they have much inner tension, I may ask if there’s psychological stress, if they feel sorrow and things like that.

C: You need to give them some tools, so they can manage their daily life better. And yes, then I’m completely into the coaching field, I give them good advice about how to deal with their lives.

The follow-ups are focused on up-dates since the last consultations and new symptoms. The therapist goes deeper into the patient’s emotional state, including psychological challenges; griefs and sorrows, tensions and struggles. The therapist adjusts further treatment according to the patient’s reactions and condition. The therapist also functions as a coach regarding specific issues.

### A good consultation is characterized by building trust

The therapists underlined that good consultations are characterized by a fruitful relationship between them and the patient. The therapist needs to be humble and show the patient respect. The patient must trust the therapist, and the therapist and the patient must have a common understanding and mutual goals.

J: I need to come across as safe, and professionally competent. Then the patient relaxes and feels that he is well taken care of.

S: The consultation is good when I feel that I and the patient are on the same level, when the patient trusts me, and tells about his problems. Some of them have a lot on their mind. And you have to guide them through to make some sense of it. And it’s when I feel that they’re relaxed on the bench, […] that I can do what I’m supposed to do. Some patients resist with all their might, and then I can’t do my job.

C: [The good consultation is] when we get the real deep flow and connection. Then I get this inner feeling of deep understanding. And I feel love for the person and for the work I do. It’s also because I know that what I’m offering will help that patient.

C: But I can’t help people if they won’t help themselves. And I tell them that. Many times I say all right I can do this and that, but this is what you have to do.

According to the therapists, the good consultation process emerges when the therapist enables the patients to speak overtly about their inner problems, and the therapist is able to interpret the patients’ statements. The process is enhanced when the patients cooperate, e.g. follow the therapist’s lifestyle advice. An optimal state is when the therapist experiences a flow and deeper psychological connection with the patient, resulting in a deeper meaning and the feeling of love. Some patients are introverted, tense, struggling against, which interferes with the process. They have to be relaxed and open up. The therapist needs to establish an atmosphere of trust and show the patients that he has confidence in himself and what he is doing, and show them his know-how.

### Healing hands and humour

The therapists characterize their therapeutic competence and strength as a combination of professional skills, showing respect and interest, self-confidence and humour.

A: In addition to knowledge and practical skills, you must be able to build trust and good relations to the patient.

C: You have to be able to analyze and understand the symptoms of the patients. And insert the correct needles and give the proper remedies. However, this is impossible to achieve if you don’t know how to talk to the patients and understand what the case actually is.

A: […] And then I may joke a little bit. I think that may loosen up difficult situations. Or I use body contact. I touch people, but I’m very apprehensive. Some patients I never touch, as I sense it’s not suitable.

J: I think my work is exciting. I can make the patients feel better by using my hands. And I’m strongly devoted to my work. I also have professional know-how, and without that, you don’t last long as a therapist. Devotion and know-how are two cornerstones in my work.

S: [….] I (have/)use body contact, I touch them, I control the treatment in a way so that I show that I know what I’m doing.

C: I feel that the energy in my hands is very good, and I use my hands a lot.

The therapists are listening, interpreting, and understanding. They build good relations. They are experienced and proficient. They show their patients respect by taking their problems seriously and being on time regarding their appointments. They may joke a bit to loosen up difficult situations. Humour is an entirely positive phenomenon and therefore a useful tool to apply in consultations. The vast majority of existing humour health research report that humour has a positive (direct or indirect) impact on health [41]. Body contact by means of the therapist’s therapeutic hands makes the patient feel confident in the therapist, and that he is in charge.

### Stimulation of the patients’ self-healing power

Patients’ positive beliefs and expectations about the treatment play a significant role in the healing process. The therapeutic setting creates a package of beliefs and expectancies towards the treatment. The more hope the therapist can bring about, the more easily the patient can start believing that it is actually possible to get well. In other words, where there is hope, there is something to work on.

S: I think that the treatment actually stimulates the healing power… That’s what I tell new students; there’s more to chiropractic treatment than two plus two equals four.

S: I think it’s much psychology. I think it’s much about how you take control of a small part of their [the patients’] lives, that you sort of control their lives while being submitted to the consultation, and that you can make them feel better. This has to do with the way you use your authority in the situation. That I firmly believe in.

C: Placebo effect is probably what we refer to as the patient’s self-healing power. That [the self-healing power] I think you stimulate in many ways during a consultation. Actually, I think from the moment you step inside the office. That you’re welcomed, that you’re seen, that we talk together, that you’re understood, that you can tell your story to a person who listens and tries to interpret and understand. And all the things we talk about to the remedy that you give. To the advice you give. And to the needles you insert. And the better all of this is, the more the patient feels that he is taken care of.

Researcher: Why do you use the placebo effect to get people better?

S: Let me put it this way. If I didn’t believe in the effect of what I do, I wouldn’t have been a therapist. But how much is placebo, and how much is the technique itself? That I can’t say. Thirty percent is the technique and 70% is all the other things we do? I don’t think you can separate those things. But I believe that if you take away one of them, it all falls apart. […] To me both parts are equally important. In other words, they are dependent on each other.

J: The examinations, the assessments we do also produce placebo […] that is, if they [the patients] have trust, then half the job is done. And when the patient feels welcomed and feels that this person [the therapist] treats me seriously, that he listens to me, then much is done.

S: And it’s obvious that much of what we do stimulate the patient’s self-healing power and that’s in everything we do. It’s the way we talk, the way we build trust, the way we touch them, everything we do strengthens the patients’ energy and self-healing power.

There is partly a specific effect of the intervention, and partly unspecific effects of the treatment, i.e. the way the therapists do things produces self-healing, i.e. contextual healing. Therapists find it difficult to distinguish between the two as both parts matter and are intertwined in the consultations. Self-healing power is similar to placebo effects.

## Discussion

This qualitative pilot study provides interesting preliminary novel insights into what complementary therapists regard as essential in patient consultations, their views of the healing process, and how the patient’s self-healing power can be stimulated.

The first consultation is extensive and includes thorough anamneses and examination. This is in line with previous research [6, 7]. During the first consultation the therapist establishes trust with the patient, typically implying common understanding and mutual goals. The therapist is in charge, exerts authority and makes the patient feel confident in the therapeutic process. The therapists contend that the patients’ positive beliefs and expectations about the treatment play a significant role in the self-healing process.

The findings in the present study are in accordance with Eyles et al. [8] who found connecting as a core theme in classical homeopathic consultations. Connecting included relationship between practitioner and patient, and the patient’s and practitioner’s engagement with holistic consultations. The homeopathic practitioners suggested that the process of connecting stimulated the healing process. Brien et al. [42] held that the consultations, and not the remedies, are associated with clinically relevant effects of homeopathic treatment. These results support the presumption that the therapeutic encounter process promotes the placebo effects, which in turn is acknowledged as the patient’s healing power [43]. Miller and Kaptchuk [24] concluded similarly and took this argument further. The aspect of healing produced by the context of the consultation, as separated from the specific efficacy of treatment interventions, is understood as contextual healing, which is “a fruitful alternative way of conceiving the placebo effect”.

Complementary therapies, such as homeopathy, are controversial since there are no plausible actions for homeopathic remedies of high dilutions, since they are ultra-molecular and no molecule of the original substance is left in the remedy. Claimed treatment effects are commonly attributed to unspecific mechanisms or interpreted as placebo effects [43]. In this context, the remedy can be understood as a ritual of administering treatment and as such a part of the interaction between the patient, therapist, and treatment environment. In such an interpretation, the healing would be a result of the clinical consultation [24]. Considering the long consultation time that the participants offered their patients, and the intensity of the complementary anamneses, it must be acknowledged that a placebo effect induced through increased attention is likely to play a role of relevant and clinical interest in complementary therapies.

Our findings show that the therapists suggest that there is partly a specific effect of the modality itself, and partly unspecific effects of the whole treatment package. According to our participants, the unspecific effects are produced by thorough anamneses, building trust, identification and agreement of a common understanding and mutual goals, being professional, taking control of the situation, and showing empathy and respect towards the patient. This is in line with Helman [23] who has made a general model describing symbolic characteristics and phases of healing rituals: The therapists may provide consistent and comprehensible explanations of cause and the kind of problem, as well as suggest how to handle the problem. In the consultations the therapists must seek consensus with the patients so that the patients get emotionally and intellectually involved in the healing process, and make whatever changes necessary.

The placebo effects appear to be considerably large in terms of chronic conditions and subjective symptoms, for which existing treatment are only partially effective in relieving symptoms [24, 44]. In a meta-analysis Hrobjartsson et al. found that the placebo effect was particularly important for conditions such as pain and nausea [45]. In the present study the patients visiting the complementary therapists had typically chronic, unspecific complaints, such as musculoskeletal pain. In the light of the healing rituals and placebo effect, complementary therapies have an integral focus: Dedicating time and building relations are essential to classify problems and find treatment. Further, the activation of self-healing power is essential for optimal treatment outcome.

### Implications for further research and clinical practice

The implementation of this pilot study demonstrated that the methods and procedures used seem feasible and may be used in a larger study with a higher number of participants and a longer observation period. Knowledge on how therapists and patients appreciate and deal with adverse effects associated with the use of complementary therapies is, to our knowledge, sparse, and we recommend that this issue is investigated in future qualitative research. In line with the results of this pilot study, we suggest that complementary therapists should deliberately elicit the placebo effect to optimize treatment outcomes in the clinical encounters.

### Strengths and limitations of the study

The findings from this pilot study should be appreciated as limited and preliminary, and be read with precaution. The short observation period in the pilot study raised potential problems in terms of observer effects and difficulties in gaining an ‘insider’ perspective. However, due to work pressure one week was the maximum time these participants could allocate to the study. Nevertheless, there are some strengths that are worth paying attention to, and may to some extent underpin the findings in this pilot. The number of participants was small. However, the considerable amount of years of clinical experience and professional skills of the therapists participating in this study may contribute to ensuring the credibility of the findings. For the purpose of strengthening the credibility, we used several approaches for data collection. Firstly, we obtained data through participant observation, secondly through individual interviews and thirdly through a focus group interview. The rationale for this approach was to elucidate the research questions from different perspectives and thereby enhance internal validity and trustworthiness of the study.

*The role of the researcher* is essential in qualitative research as the researcher is the most important tool. The researcher who collected the data, kept these issues in mind, and, if necessary, adjusted judgments accordingly. The researcher’s integrity, such as knowledge, experience, honesty, and justice is crucial for securing good quality of the research. Integrity and critical attitude towards the work were consecutively evaluated and commented on by the research team.

The researcher’s presence in the consultations may have influenced and changed the course of the consultations. This was addressed in the reflection and interviews. The therapists were asked to reflect on this issue to reveal possible differences between the observation situations and practice as usual. The two authors who did not participate in the data collection performed the analysis and interpretations.

## Conclusion

This was a pilot study. Therefore, the findings should be appreciated as limited and preliminary. According to data obtained from this study consultations were characterized by extensive consultation times, including intense, thorough anamneses. A successful consultation was characterized by mutual understanding and treatment goals between the therapist and patient. The therapists emphasized their professional skills and therapeutic competence as important when building fruitful relationships with their patients. Exerting authority and making the patient feel confident were essential factors for a successful healing process. The complementary therapists understood placebo effect as the patient’s self-healing power, resulting from establishing trust and belief in the treatment process.

## Additional file

Additional file 1: Interview Guide. (DOCX 23 kb)

## Footnotes

1 A blood-sample test to prove bacterial infection (authors’ addition)
