# Supplementary material for: "Placebo effect is probably what we refer to as patient healing power": A qualitative pilot study examining how Norwegian complementary therapists reflect on their practice
Source: BMC Complement Altern Med. 2017 May 12;17:262. doi: 10.1186/s12906-017-1770-8 (PMC5429571; doi:10.1186/s12906-017-1770-8)
Supplement: Additional file 1: — Interview Guide. (DOCX 23 kb) [file 12906_2017_1770_MOESM1_ESM.docx]

**Interview Guide**

**Interviews - Complementary therapists**

1. How did you experience the therapy today?

- Different – as usual
- How did the observer’s presence affect the consultations?

1. Differences and similarities between the first consultation and later consultations?
2. What are the most common patient complaints?
3. What characterizes good consultations? State some examples from today’s consultations, if possible.
4. Which competence is the most important for a therapist?
5. Diagnostic, technical? b) Communication – relationship?
6. What is essential to stimulate the patient’s own self-healing power? Please provide examples.
7. The connection between ”specific curative intervention” and “comprehensive healing work”?
8. What are your strengths as a therapist?

______________________________________________________________________________
